# ‘To[o] much eating stifles the child’: fat bodies and reproduction in early modern England†

**DOI:** 10.1111/1468-2281.12031

**Published:** 2013-09-09

**Authors:** Sarah Toulalan

**Affiliations:** ^1^ University of Exeter

## Abstract

This article examines associations between fat bodies and reproductive dysfunction that were prevalent in medical, midwifery and other literature in early modern England. In a period when fertility and successful reproduction were regarded as hugely important for social, economic and political stability such associations further contributed to negative attitudes towards fat bodies that were fuelled by connection with the vices of sloth and gluttony. Fat bodies were categorized as inherently, constitutionally, less sexual and reproductively successful. Consequently they were perceived as unhealthy and unfit for their primary purpose once they had reached sexual maturity: marriage and the production of children.

… the fat and fleshy habit of the man or woman hinder generation. For it hindereth them that they cannot join their genitals together: and by how much the more bloud goeth into fat, by so much the less is remaining to be turned into seed and menstrual bloud, which two are the originals and principals of generation. Those women that are speckled in the face, somewhat lean, and pale, are more given to Venery than those that are red and fat. 1

In this short paragraph the sixteenth‐century French surgeon Ambroise Paré summed up several early modern ideas about fat people and reproduction (generation in contemporary terminology) that were repeated and perpetuated by numerous other authors into the eighteenth century. Paré's work was published in English in 1634 but was available before then in Latin, French and German editions produced on the continent. 2 These ideas were not new to Paré, but also appeared in many earlier works derived from classical medical knowledge that circulated throughout England and Europe in Latin long before their translation into vernacular languages. Writing over a hundred years later, the English author and translator of a number of medical books, William Salmon, was neither the first, nor the last, to quote Hippocrates in remarking on the relative merits of different body types for successful procreation: ‘Therefore *Hippocrates* saith (speaking of the either easie or difficult Conception of Women) the first Consideration is to be had of their Species, for little women are more apt to Conceive than great; slender, than gross; … to be very fleshy is evil’. 3

Medical consideration of – and concern about – the ‘evil’ implications for health of the ‘very fleshy’ body can thus be seen not to be new, but to have a very long history. Readers can hardly fail to be aware of current anxieties about increasing obesity in U.S. and European populations (and increasingly elsewhere) and the potential impact upon a range of issues from health, life expectancy and cash‐strapped health services to food production and national security. There is a rising tide of scientific and medical research publications on the subject of what numerous writers refer to as ‘this current obesity epidemic’, including examination of problems specifically to do with all aspects of reproduction, both natural and assisted, which are indicated to increase with body mass index (B.M.I.). 4 However, the use of B.M.I. as an indicator of obesity is itself highly contested, critics noting that it is based upon a flawed formula that fails to account for muscle mass and bone density. 5 Such present concerns have further stimulated historical studies of obesity, demonstrating a very long history of negative stereotyping that continues to be seen in attitudes and prejudices today. 6 This article examines the association of fat bodies with generative dysfunction that was repeated in a wide range of medical, anatomical, midwifery and other more popular texts from the early sixteenth century to the mid eighteenth century, placing the concerns raised in historical context. It argues that these concerns were not simply an unquestioning repetition of earlier medical knowledge, citing classical authorities such as Aristotle, Galen and Hippocrates, but were shaped by the contemporary importance that was placed upon fertility and the stability (social, economic, political and religious) that flowed from undisrupted lines of inheritance. Fat bodies – both male and female – were understood to be, and represented as, inherently reproductively problematic and, as such, were disruptive bodies, resisting, through their unrestrained appetites, conformity to contemporary expectations for their gender and their class.

Generative problems that were understood to affect fat bodies encompassed a number of issues: for both men and women generative materials (seed and menstrual blood) could be defective; sexual desire might be lacking or slow to arouse, and the sexual act difficult to accomplish. Fat men were likely to experience erectile difficulties, while fat women were thought to be at greater risk of miscarriage and of suffering difficulties in childbirth, so that a pregnancy might not reach full term or result in a live birth. This list of reproductive issues that were associated with early modern fat bodies resonates with similar concerns that have been raised in modern medical research, where obesity has been linked to sub‐fertility, maternal gestational complications and miscarriage, difficult births, and post‐partum issues for both mothers and infants. 7 However, early modern perceptions of these issues have so far escaped any closer scholarly study, despite a wealth of attention to the demographic, social, cultural and medical dimensions of fertility, pregnancy and childbirth, including a more recent focus upon infertility. 8 The discussion in this article not only expands our knowledge of early modern understandings about reproduction from conception to childbirth through a close examination of these hitherto neglected issues, but also contributes to the growing debate about perceptions and representations of fat bodies in historical perspective. Early modern authors perpetuated earlier classical ideas about fat bodies that categorized them as inherently, constitutionally, less sexual and reproductively successful: they were sub‐fertile, lacking in sexual appetite, and physically unfit for reproduction, including childbirth. Contemporary thinking about bodies and sex connected sexual pleasure and desire with fertility, contributing to an erotic aesthetic in which sexually desirable bodies were bodies that were perceived as fertile. 9 These authors added to already existing negative perceptions of fat bodies (as indolent and gluttonous, lacking the virtues of moderation and hard work) by associating fat bodies with infertility, sexual incompetence and generative failure at a time when reproduction was regarded as the primary aim of marriage and a measure of a successful marital union.

Successful reproduction was also understood more broadly as important for the stability of society through the continuation of bloodlines, the successful transfer of lands, property, wealth and power from one generation to the next, and the production of sufficient bodies to serve the nation economically and militarily. 10 There was therefore also a class dimension to admonishments about moderating diet and avoiding luxurious living, with over‐indulgence in rich foods and drink, to enhance fertility and reproductive success. It was the higher classes and wealthier sections of the population who were most anxious to secure continuity of family wealth and social position, and they were also more prone to being fat as they were able to afford not only richer foodstuffs but also greater quantities of food. They also had less physically demanding lifestyles, as they did not need to labour for their living and could afford to employ servants to carry out the labour‐intensive, heavy domestic tasks necessary for the smooth running of a household without having to assist as was necessary in smaller, less well‐off households that were able to support perhaps only one maid‐of‐all‐work. 11 Authors of some medical and popular literature, such as ballads, contrasted the infertility of indolent, wealthier women who were more focused on the pleasures of luxurious living with the greater fertility of hard‐working, poor, but healthier women. 12 Such representations served to warn against disruption to the social and gender order as women neglected both their maternal destiny and their obligations to their social class. 13

Through the examination of this specific issue of contemporary understandings and perceptions of fat and reproduction this article also comments upon broader questions to do with the aesthetics of the body, and with what early modern people might have thought about what constituted an attractive and healthy body, and what bodies were for. Although it is beyond the scope of this article to examine sources that might set out the precise qualities that were thought to constitute an ideal body at this time – portraiture and literary sources among others, for example – it is nevertheless the author's contention that an important (perhaps even the most important) aspect of such an ideal body that has been overlooked was that it should be fertile and capable of producing healthy offspring that would survive childbirth. 14 Véronique Nahoum‐Grappe defines the beautiful female form at this time as ‘curvaceous’ and ‘soft’, thus more rounded and plump than slender, but Naomi Baker includes in her definition of the ugly body that it was obese; and the ugly body was also read as ‘morally corrupt’ and unhealthy. The sources examined here indicate that a significant component of a healthy body was its reproductive capacity, that it be free of all bodily impediments to successful generation. A body that was considered to have more store of fat than was necessary to make it ‘plump, equall, soft, white and beautifull’ (or not enough) was understood to be humourally out of balance and therefore likely to suffer a range of generative difficulties. Such bodies were consequently perceived as unhealthy and not fit for their primary purpose once they had reached sexual maturity: marriage and the production of children. 15 The article sets out the various ways in which bodies that were too fat were perceived to suffer impediments to successful reproduction and argues that, given the contemporary importance of fertility, a significant component of the negative perceptions of fat bodies – morally, aesthetically, erotically, medically – was informed by the adverse view of their generative potential. In particular, fat bodies were understood to divert resources (blood and nutrition) from both the production of generative materials and the nourishment of a conception in the womb during gestation to the further augmentation of the fat, thereby constructing such bodies as inherently unable to fulfil their generative or maternal role. Although not dealt with in this article (as it focuses specifically upon fat bodies) those who were regarded as too lean were also perceived as similarly generatively defective, and were invariably paired with the fat body in discussions of these issues. Questions about why negative stereotyping of fat bodies has persisted while attitudes towards, and perceptions of, thin bodies have changed over time, needs much closer investigation over the *longue durée*, with particular attention to changing attitudes as sex became decoupled from reproduction. 16

There is still a paucity of scholarship on early modern thinking about and attitudes towards fat bodies despite some excellent recent work on the subject. 17 This recent work has emerged mostly from cultural and literary studies rather than from history: historical analyses of obesity have tended to treat it as a modern phenomenon appearing in the late nineteenth century. 18 One exception is Michael Stolberg's discussion of fat and obesity in early modern medicine which argues that the pathologization of obesity is by no means a modern phenomenon but was discussed widely in early modern medical literature, together with ways of remedying it. 19 Elena Levy‐Navarro has analysed the religious and political contexts of the characterization of fat dramatis personae in early modern plays to assess negative stereotyping of the fat body and the vices with which it was associated at that time. Essays in her later collection *Historicizing Fat* have illuminated varying ways in which ‘fat is a cultural construct’, but did not include any significant discussion of the context of early modern medical understandings of the body and its humoural physiology that informed and underpinned these representations. 20 Fat was understood as an essential substance that formed a greater or lesser part of the body according to its constitution, and hence also according to sex, as the constitution associated with the female sex meant that women would have a greater store of fat than men. Thus some bodies might be understood as ‘naturally’ having more fat than others at the same time as it was also known that fat accumulated as a result of over‐feeding and a lack of physical activity. Consequently, although medical writers, too, associated fat bodies with the sins of gluttony and slothfulness, even going so far as to categorize the fat body as a deformity of magnitude, 21 they did not always do so in an entirely pejorative fashion, as the body's constitution dictated its appearance, from the colour and quantity of hair to temperament and mental acuity. 22

Although Levy‐Navarro used the term ‘obesity’ to discuss early modern literary representations of fat bodies and Stolberg uses it in the medical context, derived from the Latin ‘obœsitas’, this does not seem to be a term that was commonly used in English medical literature. In the texts examined here the word ‘obese’ occurs in only one late seventeenth‐century anatomy book, and ‘obesity’ in just one early eighteenth‐century book on the diseases of women. 23 The words ‘obese’ and ‘obesity’ as used today connote bodies that are not just overweight but that have a measurably and significantly greater quantity of fat that is associated with a range of health problems. Early modern authors usually described bodies that were fleshier than others as ‘fat’, ‘corpulent’ or ‘gross’. These terms were not used to describe or to distinguish between varying degrees of size, weight or fleshiness, but rather seem to have been used interchangeably and frequently together (‘a corpulent fat Man’; ‘Corpulent and Succulent gross Bodies’) to denote bodies that were perceived as having too much fat, without quantifying what precisely would constitute too much. 24 Pat Rogers has pointed out that before 1750 people did not tend to weigh themselves, although at least one physician had created a weighing chair in the early seventeenth century, and the physician George Cheyne in the early eighteenth century was acutely aware of variations in his weight. 25 Robert Bucholz, referencing portraiture and contemporary commentary, has indicated how Queen Anne was observed to become increasingly fatter, but there was not yet any systematic or widely accepted measurement of bodies that categorized them in varying degrees of body size or weight. 26 Fat bodies in medical texts were positioned as such only by contrast with those that were ‘thin’ or ‘lean’. How much was considered ‘too much’, or how fat was ‘fat’, is therefore impossible to quantify – although one newspaper article about a club of fat men reported that membership required a man to be too fat to squeeze through a ‘moderate Size’ door, so needing to be allowed entrance through ‘a Pair of Folding‐Doors’. 27 At this time, bodies that were designated ‘fat’, ‘gross’ or ‘corpulent’ might therefore vary considerably in size, weight and degree of ‘fleshiness’, but were consistently represented as likely to be less fertile in a variety of ways, even as some authors acknowledged that not all fat people were childless. Such bodies had to be brought to a leaner state if their reproductive capabilities were to be improved.

Through their investigations of many other issues historians have identified a contemporary concern with fertility that was also central to ideas about fat bodies. Historians of demography have demonstrated that there was a steady rise in the English population in the sixteenth and early seventeenth centuries that came to a halt in the mid seventeenth century, when it began to fall again. 28 This dip in growth was explained by several factors: the late age of marriage; high infant mortality, where one child in four died before the age of nine; high mortality in the population more generally as a result of endemic diseases that carried high mortality rates, such as plague, typhus and smallpox, and of war, especially the English civil wars; and relatively high levels of emigration to the West Indies and the North American colonies. It has been argued that there was therefore a perceived need to enhance and preserve reproductive health to ensure there were more healthy pregnancies that might result in more surviving children. 29 Population growth resumed in the early eighteenth century as the age at which couples married began to fall, allowing a longer period of marital fertility, which was noticeable from the mid eighteenth century. 30 Concerns with fertility and ensuring a healthy pregnancy, though, are apparent in writings on the subject both pre‐ and post‐dating this period of population stagnation, suggesting that successful reproduction was regarded as important in English and European societies where life might always be precarious in a world of endemic and epidemic fatal diseases, of recurring conflicts leading to fatalities at local, regional and national level, and of bad weather leading to harvest failure and famine. Prescriptive texts and church teachings also promoted procreation as the primary aim of marriage, ensuring the next generation of Christians and binding couples together in mutual support and endeavour to care and provide for their children. Social, economic and political stability depended upon inheritance and the smooth transfer of titles, money and land down through the generations, as well as on the availability of sufficient bodies for work and military strength. 31 Successful generation was therefore of enormous importance throughout this period, promoted through a variety of contemporary media, and longed for by childless couples.

While historians of demography have long studied patterns of fertility to explain changes in populations over time, historians of medicine and sexuality have only more recently begun to pay attention to early modern ideas and concerns about fertility and barrenness. Earlier scholars paid attention to barriers to fertility, particularly knowledge of and recourse to contraception and abortion, and to the impact of unwanted pregnancy and illegitimacy. 32 Researchers are now investigating more closely the meanings and significance of both childbearing and childlessness in early modern societies. Laura Gowing has noted the importance of pregnancy and childbearing to women's lives, not only because it marked their full transition to womanhood, but also because it conferred status within the community and the communal areas of female life with the knowledge of sex and reproduction that it brought. 33 Helen Berry and Elizabeth Foyster have argued that fatherhood was also important to early modern men and how perceptions of impaired fertility affected their social standing and reputation, while Jennifer Evans has suggested that knowledge of and demand for remedies for barrenness and impotence were widespread. 34 However, while some historians have noted briefly that fat was regarded as one cause of infertility there has not been any in‐depth examination of exactly how fat was understood to interfere with generation, nor what remedies for infertility from fat were advocated by physicians. 35 This article rectifies this omission and argues that the perceived impaired fertility and maternity of fat bodies also contributed to their negative stereotyping, particularly as at least some authors regarded it as a problem that was difficult to overcome because those who were fat were likely to be resistant to becoming more lean.

Writings on generation in English, available to a lay and professional English readership, were not confined to English authors, but also consisted of a wide array of translations of European‐authored works, both from vernacular languages and from Latin, the international language of knowledge and culture. Writers read and borrowed from each other, circulating not only ideas but also words, phrases, sentences and sometimes whole paragraphs in a shared culture of medical knowledge, learning and innovation. Although primarily included in medical and midwifery texts, information and advice about reproduction and childbearing also appeared in other sources more widely accessible to a non‐medical audience, such as ballads, chap‐books, almanacs and other less‐specialized texts. Records of book ownership have also indicated that some medical and midwifery books were possessed by men and women without professional medical training or qualifications, suggesting that such works were not only the preserve of a specialized readership confined to the medical professions. It is thus likely that many of the ideas uncovered here were not restricted only to those who practised medicine and midwifery, but had a wider circulation, however generalized and simplified for the non‐specialist.

One historical continuity in the discussion of the impaired fertility of the fat body is the relative attention paid to gender: study and analysis of problems with conception in women predominate in this field of medical and scientific investigation. 36 It is perhaps inevitable that more attention was paid to the female body as it is the one in which conception either does or does not take place; and it is, of course, the only body in which problems of gestation and parturition may occur. Historically, the female body has been ‘naturally’ identified with reproductive function and the subsequent nurturing of children; motherhood has been regarded as central to female lives, both as successfully achieved in establishing a family (legitimate or illegitimate) or as reproductive failure in childlessness. 37 This gender difference is also apparent in early modern medical discussions of reproductive problems caused by the fat body, where those arising from the fat male body were comparatively less frequently mentioned, though not entirely absent. Negative attitudes towards fat bodies, and particularly fat female bodies, may have been further bolstered by the perception that they resisted expectations for the fulfilment of gendered social roles: that men should be active, virile patriarchs able to exercise self‐mastery over appetites and behaviour, and that women should marry and become mothers. 38

Bodies were understood to be both fat and to experience generative difficulties as a result of humoural imbalance. The imbalance that produced excess flesh in the body – too many cold and moist humours – also caused reproductive disorders in both men and women, affecting the production of seed or semen and menstrual blood, erectile function, and the arousal of sexual desire. Medical writing about fat in the body was generally consistent throughout the two centuries from *c*.1550 to 1750: as will be apparent from the following discussion, eighteenth‐century writers repeated the words and ideas – sometimes almost word for word – of much earlier sixteenth‐century authors such as Eucharius Röesslin, Levinus Lemnius and Ambroise Paré, in a manner of writing that was typical of this period. Such borrowings ensured that the influence of these early authors stretched well beyond their lifetimes, even as earlier knowledge was usurped by new discoveries and theories. Ideas about chemical medicine, for example, were frequently incorporated into a humoural framework, while into the eighteenth century references to eggs and ovaries gradually replaced those to female seed. 39 Nevertheless, medical books continued to repeat the same kinds of statements about the nature and purpose of fat in the body, and about what kinds of bodies were likely to have more fat than others as a result of their humoural constitution. 40

Bodies were understood as composed of four humours – blood, yellow bile, black bile and phlegm – which had four qualities – hot, dry, cold and wet. The constitution of a body was related to its particular balance of humours, with men more naturally hot and dry, women more cold and moist. There was some discussion in medical books as to whether fat should be understood as hot or cold in nature as its composition might change from solid to liquid depending on its temperature. Thomas Vicary in 1577 noted that ‘The flesh is a consimiler member, simple, not spermatike, and is ingendred of blood congeled by heate, and is in complexion hote and moyst’. 41 Helkiah Crooke, in *Microcosmographia* (1615), however, wrote that Galen determined fat to be congealed by cold, but also set out the arguments of those who concluded the opposite, noting: ‘Those that hold the contrary, do thus demonstrate the matter of Fat to be hot, the worker of it heat, and the effects of it hot’. 42 Although some authors, like Crooke, observed that fat might have both hot and cold qualities, ultimately it was categorized as cold, being congealed in the body from ‘the coldness of the Membranes, from whence it gets its white color’. 43As Vicary had stated, fat was understood to be made from blood. Crooke described the fat as ‘bedded betwixt the skinne and the fleshy membrane … It is ingendered of the more oylie, thinne, and ayrie portion of pure and absolutely laboured and concocted bloud, distilling like a dew out of the smal and capillarie veines of the habite of the body’. 44 Over half a century later, William Salmon described it in similar terms: ‘*Flesh* and *Fat* are made of Blood alone; *Fat*, being according to *Riolan* … the thinnest substance of the Blood; Oyly, Sweating out through the tender Coats of the Veins, and hardning between the Membranes’. 45 Although couched in more precise anatomical language and description later in the eighteenth century, authors’ understanding of its nature can be seen not to have changed fundamentally: ‘It is *in Substance a gross whitish Oyl*, and is indeed nothing but the *Oyly* part of the *Aliment*, separated from the *Arterial* Blood by the *Adipose Glands*, and carry'd by peculiar *Ducts* to the *Membranous Cells*, from whence it is transmitted to the Blood again by the *Veins*’. 46 Fat was thus understood as a basic and necessary component of the body, albeit one that was of a lesser quality than others such as the brain, heart or liver, serving several important functions such as improving appearance by filling up the empty spaces of the body, cushioning the parts for comfort, greasing the joints to make them more supple, keeping the body warm, and providing nourishment in times of famine. 47

As fat was congealed in the body by cold those with a colder constitution had more fat than those who had a hot constitution, and as women were constitutionally more cold and moist than men, women naturally had more fat in their bodies. According to Crooke, ‘If we consider the habite and structure of the parts of both Sexes, you shall finde in men more signes of heate then in women. The habit of a woman is fatter, looser and softer’. 48 Contemporary understandings about the important role of heat in generation meant that colder and fatter bodies were regarded as potentially infertile, barren bodies. Samuel Haworth defined those of a cold and moist, or phlegmatic, constitution as having very little appetite for sex: ‘The next is the Phlegmatic Constitution; they of this Temperament are Cold and Moist, the colour of their Skin is white, they are fat and obese … and have very little Inclination to Venery’. 49 Thomas Bartholin's conclusion that ‘The colder Creatures are the fatter, as Gueldings, Females’ neatly links these ideas as his first example of a cold fat body is of a creature that has no generative ability. 50 Through the juxtaposition of ‘Gueldings, Females’ he also implicitly places responsibility for reproductive failure onto the colder, fatter bodies of women. Women, then, as more naturally fat, were more likely to be considered at fault when a couple were unable to reproduce.

In the humoural model of the body a satisfactory balance of the humours was essential for conception to take place. Authors advised that too much heat, cold, dryness or moistness in the generative parts, particularly in the humoural balance of the womb for women or the nature of the seed in men, could prevent procreation. Eucharius Röesslin in *The Byrth of Mankynde* in the early sixteenth century wrote that

yf the matrice be distempered by the excesse of any of these foure qualities then must ye reduce it agayne to temperancie by suche remedyes as I shall shewe you hereafter. Lykewyse maye there be defecte and lacke in the man as yf the seade be ouer hote the which the woman shall feale as it were burning hote or to cold the which he shall feale as it were in maner colde. 51

In the mid eighteenth century the French physician and professor of medicine Jean Astruc continued to write in humoural terms about encouraging successful procreation, where ‘very cold, languid and insensible Women’ would not conceive and should be treated with remedies that were heating. 52 Heat was a crucial ingredient in both sexual pleasure and successful conception; raising the body's heat was essential to raising desire. The friction of intercourse raised heat leading to orgasm, and, for a woman, the special properties of semen itself, which had vital heat in three elements, might further raise her sexual heat, encouraging conception. 53 Heating foods, herbs and wines might arouse lust in both men and women, as could the application to the genitals of oils and ointments with heating properties. 54 Books such as *The Countrymans Physician*, aimed at self‐treatment of common ailments of each part of the body, began the section on curing barrenness with remedies aimed at rectifying barrenness from cold. 55 Cold and moist humours and lack of heat in the generative parts causing infertility and absence of desire were thus associated with the fat body.

Cold, fat bodies were likely to be less fertile because they were unable to produce seed of good enough quality or quantity to allow conception to take place. This was because blood was used to produce fat, leaving less available to produce seed (as Paré had commented, ‘by how much the more bloud goeth into fat, by so much the less is remaining to be turned into seed’), and what seed it did produce was likely therefore to be of poor quality. 56 The poor quality of the seed itself was then a cause of reduced desire for sexual intercourse so that either intercourse was avoided altogether, obviating any possibility of reproduction, or a man might experience weak, unsatisfactory erections preventing completion of the act of intercourse. In *The Problemes of Aristotle* (1595) several questions and answers on the subject of generation and what might prevent it provided explanations for the barrenness of those who were fat. Some of these focus upon the quality and quantity of their seed. It was assumed in one of these questions that barrenness proceeded from an insufficient quantity of seed produced during intercourse:

*Why do such as are corpulent cast forth little seed in the act of copulation, and are oft barren?*

Is it because the seed in such passeth into the nourishment of the body? And for the same cause, corpulent women have but small store of flowers. 57

Neither the question nor the answer here is gender specific on the question of the production of seed, so may be interpreted as referring only to men as seed‐producers in the Aristotelian model of reproduction. In this model men produced seed for conception while women furnished only matter in the form of the menstrual blood (‘flowers’) that was shaped by the active principal of the male seed to form the foetus, and which was also retained during pregnancy to provide nourishment to it in the womb during gestation. However, as this is not explicitly stated, a reader might also interpret it in light of the Galenic‐Hippocratic model of generation in which both men and women ejaculated seed at orgasm which mingled to form a conception, although the woman's seed was thinner and more watery than men's, and hence ‘a more imperfect seed’. 58 Women, as the colder of the sexes, produced colder seed that required men's seed infused with vital heat to form the new life, as Jane Sharp noted in her *Midwives Book* in 1671: ‘Mans seed is the agent and womans seed the patient, or at least not so active as the mans’. 59 Both these models were prevalent in medical writings on generation and in more popular literature throughout the early modern period and into the eighteenth century, and this ambiguity would allow readers to interpret the answer within whichever model informed their understanding.

Fat and seed for procreation were both made from blood. Crooke called seed ‘The froth of the best and most laudable blood’, while John Marten in the eighteenth century noted that ‘the *Seed* of Man is made of the best Arterial Blood, sent to the *Seed*‐Vessels from all parts of the Body to be there elaborated’. 60 Therefore, if more fat was produced from the blood in the body there was less available for the production of seed. This logical deduction was presented in writings on reproduction from the sixteenth century into the eighteenth: ‘*Aristotle* dooth affirme, that euery fat creature hath but small store of seede, because the substance of it doth turne into fatnes’; and John Marten elaborated two hundred years later, ‘in Fat People, part of that Blood which should go to the making of *Seed*, turns into Fat, whereby the *Genital Parts* are depriv'd of that quantity, and of that Spirit and Strength which is requir'd to quicken the *Seed* and make it fertile’. 61 ‘Defective’ seed was thus both deficient in quantity and in quality, being ‘vitious, or unfit for Generation’. 62 Matter which should have been used by the body to make seed for generation was diverted in fat bodies into the production of flesh, thus reducing both its quality and its potential quantity.

Seed, as explained by the Dutch professor of anatomy Isbrand de Diemerbroeck, was concocted in a complicated process from the blood into ‘*a frothy Liquor*’ whose composition was mainly salty, from which derived ‘its fruitfulness and balsamic Power’ and the term ‘salacity’ for lust. Thus, ‘in drier Bodies, where salt Humours predominate, much Seed is generated, which makes ’em more able for the Sports of *Venus* … Because the increasing of that in quantity excites an itching Titillation, and provoke to Lasciviousness’. 63 In contrast, bodies in which there were fewer salt humours, such as fat bodies, produced less seed, so had less desire for sex and were consequently less fertile: ‘Because in fat Bodies, where fat and sulphurous Humours predominate, there is little Seed generated, and hence they have little proclivity to Venus’. 64 Perhaps not surprisingly, as he had translated Diemerbroeck's *Anatomy*, William Salmon in his *Ars Anatomica* published in 1714 repeated the same explanation for why fat bodies produced less seed and were less interested in sexual activity. 65 This lowered level of desire for sex resulting from a man's defective seed thus also meant that a man was likely to have poor erections, further incapacitating his generative ability. The Dutch writer Levinus Lemnius in the mid sixteenth century had explained that

because their Sperme is too thinne and moyst … this moystnes & humour is slowly forced forward by heate, and the members of genereatio[n] not filled w[yth] swelling spyrit, it foloweth th[at] they unto carnall coiture fu[m]bling, slow, & not greatly therto addicted, neither therein take anye greate delectacion or pleasure. 66

One hundred and fifty years later John Marten also noted that fat men, having weak and infertile seed, would also have weak erections that ‘are not so frequent, nor altogether so potent as before he arriv'd to that fatness’, so that even if he managed coitus ‘it is seldom found that the *Seed* is prolifick, or any thing comes on that conjunction’. 67 As ‘prolifick’ meant ‘full of blood’ Marten was here alluding to the diversion of blood from producing seed to augmenting fat, diminishing its reproductive quality so that intercourse would not result in a conception. Thus not only was infertility a consequence of weak seed that was unfit for generation, but the diminution of generative power attributed to the seed also had an effect on a man's desire for sex, and hence on his erectile function. A fat body tended to produce more flesh than generative material. It was thus an inherently less sexual body, diminishing a man's virility and undermining his manhood. 68

As we saw in the quotation from Paré cited at the beginning of the article, there were two essential components for generation: ‘seed and menstrual bloud, which two are the originals and principals of generation’. In the same way as fat was a cause of defective seed, it also had a negative effect on the menstrual blood, hence further impairing women's fertility. As was noted in the *Problemes*, women who were fat might also have only a ‘small store of flowers’, that is, very little menstrual blood. Although it was not elaborated upon further in the *Problemes*, other authors touched upon how the menstrual irregularities of fat women might impede their fertility. In both Aristotelian and Galenic‐Hippocratic models of generation the menstrual blood played a role in conception and gestation, providing matter to the foetus and then subsequently nourishing it within the womb. The establishment of a regular and moderate flow was therefore also crucial to successful reproduction, and women (and physicians) were concerned to regulate this aspect of women's lives in order to optimize their fertility. Cathy McClive has noted the importance of establishing a regular menstrual flow in a girl's transition to womanhood, while Susan Broomhall identified this concern as one that was key to deciding whether or not a girl was ready to commence a regular sexual relationship and that she would thereby be able to conceive. 69 Nicholas Culpeper in the mid seventeenth century asserted that menstruation was likely to be disrupted in a fat woman: ‘Again, Many times they are stopped in immoderate fat people, for their Veins are narrow, and that little Blood they have is turned into Fat’. 70 Fat both caused an obstruction in the body, impeding the expansion of the veins for the blood to flow through, and it diverted blood from generative matter to its own creation or nourishment. A fat woman's fertility was thus also impaired because she would be unable to establish a regular menstruation which would indicate her readiness for conception. Although not explicitly stated here, a further implication was that, should a conception nevertheless take place, there would be insufficient menstrual blood produced for the nourishment of the growing foetus in the womb, because in the same way as less blood was available to produce seed, so there was less to form menstrual blood. The fat female body was therefore also likely to have insufficient or defective generative matter, reducing its maternal potential.

In addition to the negative effects of too much fat on generative matter – seed and menstrual blood – and sexual desire, many authors also identified the excess flesh of the fat body itself as an impediment to successful intercourse. Philip Barrough in *The Methode of Phisicke* (1583) stated that those who wished to have children should be careful in their diet so that they did not become fat as this would prevent intercourse as well as reduce seed production: ‘And you must specially obserue in their order of good diet, that neither the man nor the woman be made fat. For they that be fatte, are vnapt to procreate and beget children, because their genitours can not touch together, and also because they send out little seede’. 71 The excess flesh of the body of one or both partners would simply prevent procreation because it would obstruct penetrative sex, either fully as suggested by Barrough above, or partially as penetration might not be as deep into the vagina as was needed to cast the seed into the womb. As Barrough further noted, ‘for fat men haue such great bellies, that they cannot cast the seede into the deepest partes of the bodie’. 72 Jacob Rueff, writing in the mid sixteenth century in Latin and German and first published in English translation in 1637, identified the loss of vigour in the seed, caused by having to travel too far after ejaculation when bodies did not fit closely together during intercourse, as a barrier to conception:

*Aristotle* attributeth this disability and impotency principally to fat men and women, because of the evill proportion, and ill disposition of the generative members, that is to say, in whom the seed is procured and derived from a more remote place, and so vitall spirit inclosed in it doth vanish away sooner by that delay. 73

As we have seen, in fat bodies seed for conception was likely to be of insufficient quantity or defective as blood was diverted from seed‐production to the augmentation of the fat. But it could also lose its ‘vitall spirit’, and so become useless for conception, after ejaculation because body fat prevented its easy and swift passage to the womb.

An over‐abundance of fat meant that both male and female bodies were poorly suited for the act of sex as a great store of flesh made intercourse and the ejaculation of male seed into the female body difficult to achieve. But a fat woman's body presented yet another impediment to conception should the seed nevertheless manage to reach the womb's entrance. Throughout this two‐hundred‐year‐period writers on medicine and midwifery repeated Hippocrates’ observation that in fat women the omentum (also termed the cawl 74 or epiploon – tissue connecting the stomach with the liver, spleen and colon) could compress the womb preventing the seed from entering it. The omentum was identified by writers on anatomy as a part of the body that was by nature fatty, so that in those who were fat it would have an even greater store. Thomas Browne in *Nature's Cabinet Unlock'd* (1669) described it as ‘a double membrane, arising from the Peritoneum, inter‐woven with many nerves and arteries, and covers the ventricle and intestines … it is called with the Greeks, Epiploon, because of its fatness with which it overspreads the belly’. 75 Lorenz Heister in 1721 described it as ‘a membranous Part, generally furnished with Abundance of Fat’. 76 In *Aristotle's Masterpiece* (1684) it was further noted that it was the greater storage of fat in this part that was a direct cause of barrenness in women because it prevented the seed from entering the womb: ‘when the Woman grows fat, so that the Caul swelling and bearing beyond its Bounds with its fatness, obstructs the Passage into the Womb’. 77 This cause of barrenness continued to be remarked upon as particular to fat women in the eighteenth century, even by authors, such as Henry Bracken, who were beginning to reject some aspects of classical understandings of reproduction. Bracken in his 1737 treatise on midwifery repeated in very similar phrasing what earlier authors had said about the omentum and barrenness in women:

*Sterility*, which is often occasioned … from some Part which compresses and squeezes the Neck of the Womb to such a Degree, that it cannot open to receive the Seed, as from the *Omentum* or Nut, as ‘tis commonly called in fat People, pressing closely the Neck of the Womb. 78

However, the impediment to sex and conception that was presented by fat was not considered to be a permanent one, and authors presented two possible remedies: changing diet and/or sexual position. Crooke had commented in the early seventeenth century that fat, ‘by streightning the mouth of the wombe becommeth an ordinary, but yet not a perpetuall cause of barrennesse or sterility’. 79 The French *accoucheur* François Mauriceau, later similarly asserted that female barrenness from fat could be temporary if the woman became thinner: ‘Women exceeding fat do not conceive, because the Cawl compresseth the orifice of their Womb, neither can they till they grow lean’. 80 The French physician Lazarus Riverius was more direct about what must be done to cure this type of barrenness:

Over great Corpulency, must be corrected by an extenuating Diet and convenient Evacuations. If Barrenness seem to arise from a bad Course of Diet, as in persons given overmuch to Belly‐cheer, to Wine, or small Drink, such women are to be reduced to an exact Course of Life; and all excess of eating and drinking must be avoided. 81

These comments on over‐eating and drinking and on the kinds of foods consumed were more likely to apply to the middling sorts and upper classes of society – those who were also more likely to be able to read the literature of health advice – than to the poor whose diets were more restricted and who laboured for a living. 82 Such a course of life also ran counter to the general principle of moderation for good health, and intimated that practice did not always heed prescription.

Mauriceau also – reluctantly – pointed out another possible remedy for overcoming the impediment caused by too much fat stored in the omentum: changing sexual positions. He wrote: ‘I do not willingly admit amongst the causes of barrenness this compression of the inward orifice by the *Epiploon*, forasmuch as *Aritin* hath very well remedied it, by some of the postures invented by him, by which this orifice need not be so compressed in the action’. 83 Although Mauriceau did not specify which positions might be favoured, readers, particularly educated gentlemen, would be likely to recognize the reference to Aretino's postures as suggesting sexual intercourse in other positions than the approved one of man on top and face to face. 84 Writing around twenty years later the French physician Nicholas Venette was more explicit about how those who were fat might engage in sexual intercourse and conceive. In his *Tableau de l'amour conjugal*, translated into English in 1703 as *Conjugal Love Reveal'd* and hugely popular, going through numerous editions in the eighteenth century, Venette advised that when a woman was so fat that her stomach was an impediment to sexual intercourse with her husband lying on top of her, thus preventing conception, he might penetrate her from behind instead: ‘And ’twould certainly be more advisable to put a Man upon Caressing his Wife the back way, than insinuate a dissolution, particularly when the Woman is naturally fat, and her Belly so picked, as to baffle the efforts of the common Situation’. 85 Venette went on to argue that for similar reasons, ‘When a Man is too heavy, and the Woman extreamly tender, I am of Opinion that ‘tis not contrary to the Laws of Nature to Caress sideways in imitation of Foxes’ because ‘One may by this Posture avoid all the Accidents a tender Woman is exposed to in the common Situation, and no Suffocations or Miscarriages ever happen thereupon’. 86 Venette was clearly slightly uneasy about recommending such positions without qualification as they went against the usual teaching about the ‘proper’ position for intercourse, but justified doing so by arguing that these were positions that promoted conception, this being the primary aim of marital sex:

The Matrix of Females is in a better condition to receive the Seed of the Male, and better disposed to retain and foment it, it not being able to slide out so easily as in another Posture; and Experience having confirmed to them, that Woman [*sic*], before barren, have been impregnated after this manner. 87

Like Mauriceau and Venette, Henry Bracken too advocated changing sexual position, ‘by putting the Woman in a proper Posture’, though without specifying how, as well as recommending ‘a spare Diet and low living to fat People, if they expect to be fruitful’. 88 Bracken's language here about expectations of fertility related to body size hints at a shared common reproductive knowledge and perhaps a judgemental attitude towards those who were fat and therefore responsible for their own infertility.

Bracken's attitude was perhaps influenced by his recollection, as he reflected back on his practice, that of all those he had treated for inability to conceive, sometimes over many years, his remedies had failed only in the case of those who were fat. Somewhat reluctantly, Bracken seems to lay the blame more at the door of the husband, who was very fat indeed: ‘I only remember two in which it failed, which, perhaps, might be a Fault in the Husband; for one of them was exceeding corpulent, weighing at least twenty Stone, and she none of the leanest of Women’. 89 Bracken here quantified the husband's size, estimating his weight as indicative of his ‘exceeding’ corpulence. While many authors suggested that infertility from fat was something easily reversed as people could simply follow a ‘spare diet’ to bring themselves back to a better ‘habit of body’, Bracken was less optimistic of success, commenting that ‘Fat People are unwillingly perswaded to live so sparingly, as may bring them to Leanness; if they would do so effectually, they would then be convinced that their former Bulk was the occasion of their *Infecundity*’. Such comments also imply that this was a fertility problem that applied to a certain class of people, those who could afford the quantities and types of food that would produce such ‘exceeding’ fatness of body and who could then exercise choice in moderating their lifestyle. Bracken was also forced to admit that a fat body was not a certain cause of barrenness, but countered this possible objection with his final comment that ‘yet some Exceptions strengthen rather than weaken general Rules’. 90

Medical, anatomy and some midwifery texts set out, discussed and debated the newer theories of egg‐producing ovaries in women and ‘animalcules’ in sperm that had been revealed through investigations by William Harvey, Reinier de Graaf, Marcello Malpighi, Antonie van Leeuwenhoek and others in the seventeenth century, but the concepts of heat and cold and of superior male seed were still deployed to explain reproductive success and failure. 91 Authors altered their terminology to reflect these new discoveries, referring to ‘ovaries’ in women rather than to ‘testes’, for example, but also continued to quote from the classical authors and to reiterate ideas that had appeared in sixteenth‐century texts. William Cheselden made no mention in the first edition of *The Anatomy of the Humane Body* (1713) of the potential obstruction to conception caused by the omentum in fat women, but by the third edition, he had expanded the discussion to include speculation that fat may obstruct the Fallopian tubes, so preventing conception:

I have seen in a woman both the Fallopian tubes unperforated, which upon the foregoing hypothesis, must have caused barrenness … and perhaps the fat in the membrane that connects the Ovaria to the tubes, may in very fat women, so keep these tubes from the Ovaria as to interrupt impregnations. 92

Similarly, in letters published from his practice the following year, Dr. Carr noted, in a letter advising a patient on promoting fertility, that fat obstructed conception. The patient and his wife had clearly also consulted Carr in person as well as by letter, as Carr was able to dismiss this possibility in their case: ‘3*rdly*, Some are barren by their being too fat, for too much Fat about the *Tubes* and *Testes*, hinders them in their proper Offices. There is no need of any Considera[t]ion in this Case, because it is far from being your Wife's’. 93 Although the terms used for the reproductive parts had altered, nevertheless understanding about the way that fat prevented conception remained the same.

Should a fat woman overcome all these obstacles to conception she was yet likely to remain childless because fat was also understood to be one of the reasons for miscarriage, or abortion (‘abortment’) as it was usually designated at this time. The term thus carried within it the possibility of the expectant woman's deliberate ending of her pregnancy but in the context of these writings about barrenness it invariably meant involuntary rather than elective termination. Authors frequently observed that fat women were more likely to miscarry. Röesslin at the beginning of the sixteenth century defined miscarriage thus:

Aborcement or untymely byrth is when the woman is delyuered before due season & before the frute be rype: as in the. iii. iiii. or. v. monethe before the byrth haue lyfe and sometymes after it hath lyfe it is delyuered before it steare beynge by some chaunse dead in the mothers wombe. 94

The first cause of miscarriage he listed was that the mouth of the womb did not close after conception, or that the womb was so slippery that the conception could not be retained; the second he attributed to the state of the woman's body. If a woman was too fat, the foetus was likely to die of starvation in the womb because the passages through which its nourishment travelled would be obstructed, or they might be so swollen that they would break. The

cotilydons that is the vaynes and synnues by the which the co[n]ception and feature is tyed and fastened in the matrice (through the which also the feature receaueth noryshment and fode) be stopped with vyscous and yll humours or elles swollen by inflation so that they breake by the whiche meanes the feature destitute of his wont noryshment peryssheth and dyeth. 95

Alternatively, if not starved of sufficient nourishment, the growing foetus could be suffocated by bad nourishment from too much badly digested rich food: ‘excesse fedynge and surfetynge by the which the byrthe is suffocat and strangeled in the bellye and the fode corrupte for lacke of due digestion’. 96 Rueff in *The Expert Midwife* also listed an intemperate appetite as harming the foetus: ‘over much repletion and surfeting, the waies and passages of nourishment being soone stopped, doth suffocate and choke him’. 97

Mauriceau also repeated the idea that in fat women, the cotilidons being swollen by ‘ill humours’ – or in his words, ‘viscous filth’ – would break, starving the child of nourishment. But the breaking of the cotilidons could also release the child from the womb, causing miscarriage: ‘any Woman indifferently corpulent, that miscarries the second or third month, without manifest or apparent cause, it is, because the Cotylidons of the Womb (which are the inward closures of its vessels) are full of viscous filth, by reason of which they cannot retain the weight of the *Foetus*, which is loosened from it’. 98 The author of *Aristotle's Manual of Choice Secrets* at the end of the seventeenth century repeated the same ideas but shifted the focus from the woman's body being unable to bear the weight of the child, and so losing its burden, onto the growing child, who is so over‐fed that it is unable to hold on and retain its place in the womb, being ‘so unweildy it cannot keep in its Place, and therefore is constrained to come forth before it's [*sic*] time’. 99 A few authors took up Röesslin's first point that if the womb was too slippery the foetus could not keep its place and related this to a woman being too fat, but without any further explanation. Culpeper stated that ‘Fat Women are subject to Miscarry by reason of the slipperiness of their Wombs’. 100 Not surprisingly, as she borrowed from Culpeper, Jane Sharp also observed that ‘fat women have slippery wombs’ and so were not ‘fit for child‐bearing’. 101 In the humoural model it was obvious that the excess cold, moist humours that were characteristic of fat bodies caused the womb to be slippery; it may also have been thought that as fat was an oily substance, consequently the wombs of fat women were likely to be extremely slippery. In this context, we might speculate that the multiple miscarriages of Queen Anne in the later seventeenth century may have been understood at the time as related to her increasing corpulence. 102

These ideas were repeated throughout the seventeenth century and into the eighteenth, sometimes with some further elaboration, sometimes reproducing the exact text of *The Byrth of Mankynde* or from Thomas Raynald's later version, sometimes abbreviating it. Jacques Guillemeau in *Childbirth* briefly noted: ‘To[o] much eating stifles the child’. 103 Peter Chamberlain simply reproduced Röesslin's text. 104 William Drage re‐phrased it, observing that one of the second causes of miscarriage is ‘Fatness of the *Uterus*, whose Bodies also usually are fat’. His fifth item, ‘Nourishment’ as a cause of miscarriage, therefore also included ‘From too great plenty of it, as surfeiting of too much Meat and Drink, strangles it’. 105 In the eighteenth century the same advice about diet prevailed, with John Maubray reiterating: ‘VICTUALS; if taken *too much* at a time, suffocate the *INFANT*’. 106 Maubray, however, further argued that in a woman who over‐ate the nourishment would be diverted from the foetus to the woman herself, just as blood was thought to be diverted from the production of seed and menstrual blood to the fat: ‘A *nimious* and too great an *Obesity* or *Fatness* … converts the *CHILD*'s *Nourishment* to itself’. 107 The fat body diverted all nutriment that should have been used for generative purposes to maintaining and augmenting itself, whether it was from the production of seed or menstrual blood, or nourishing the child in the womb, making it a body that was unfit for generation. The female fat body was thus characterized in reproductive theory as an inherently un‐maternal body, diverting resources to itself rather than to fulfilling the woman's primary purpose of producing and nurturing the next generation. Medical and midwifery books thus contributed to the regulation and disciplining of women's bodies and reproductive function, admonishing them to moderate their diet and lifestyle not only as a basic moral principle of health in the Galenic regimen, but also specifically to optimize their generative potential. 108 Fat women could be regarded as subversive, even scandalous, because they literally embodied a rejection of what was understood to be their primary purpose and social role. 109

Like infertility caused by too much fat, miscarriage might be prevented if the woman ‘absteyne for a tyme’ so that she reduced her body fat, removing these impediments to a successful gestation. Some authors advised that a pregnant woman could aid her weight loss with ‘some easy & gentyll medicine which may alleuiat and lyghten her of her surfetynge burthen’. 110 Recipes for such medicines were included in medical and receipt books, enabling readers to make their own for consumption (and implying that they should), such as the following: ‘*A Drink to make the body lean*. Take of round Birthwort one dram, the lesser Centory one scruple, Gentian, Poly, Parsley, each three drams, powder them and take them with white Wine fasting’. 111 In Raynald's version of *The Byrth of Mankynde* he added to this ‘especially by vomitting’, which if not achieved by drinking half a pint of water with honey, ‘with her finger or with a feather put into her throate, let her prouoke her selfe to vomit’. 112 However, Jane Sharp contradicted Raynald's advice to use a gentle medicine to induce vomiting with the firm statement that ‘purging, especially in the first, or second, or about the last months, and vomiting is far worse’ as it might itself cause miscarriage, and should therefore be avoided. 113

In writing about childbirth authors repeated ideas that fat bodies caused hard labours and more problematic deliveries. Labouring mothers were understood to have harder and more painful labours if they were fat for two reasons, both of which originated in excess fat as the cause of obstructions to bodily functions. Just as great quantities of fat in the body pressed upon the internal reproductive organs preventing seed from entering or slowing down its passage to the womb, so also birth could be obstructed as fat closed up the opening from the womb or squashed the birth passage, making it too ‘streight’ or narrow for the child to pass through. Fat women therefore required intervention to allow the child to be delivered: the midwife would have to manually open and dilate the passages so that the child could be born. Furthermore, all pregnant women were understood to have more difficulty catching their breath than women who were not expecting a child, but in fat women this problem was exacerbated as fat further constricted the breathing passages. This meant that fat women were less able actively to assist delivery by pushing the child out because they did not have as much stamina as other women. Both these issues appear in one man‐midwife's published case histories from his practice in the early eighteenth century, suggesting that this was not simply theoretical knowledge that had been repeated as a truism through two centuries of writing on pregnancy and childbirth, but that such problems were occasionally encountered in reality.

Writers on midwifery were concerned about women suffering very much during labour because it might not bode well for either mother or infant: ‘Hard‐Travail, is of it self dangerous; in which sometimes the Mother, sometimes the Child and sometimes both do loose their lives’. 114 Any obstruction to the birth of the child was a serious problem in childbirth: if the child became stuck and could not be delivered, it would die, resulting in a stillbirth, or be so weak when eventually delivered that it died soon after. If the child became stuck it might be decided that, as it would die anyway, the mother's life could be saved by dismembering the child to enable its extraction. Leaving the child inside the mother's body was a sure death sentence for the mother as she would die of infection as the child putrefied, but extraction by dismemberment or caesarean section was also risky. 115 Midwifery casebooks, such as those by Sarah Stone, Edmund Chapman and William Giffard, recorded numerous occasions in which babies died during the birth, or soon after when the labour had been long and arduous, or were killed in the process of extraction, and of mothers who died during and after long, hard labours when the baby could only be brought out with extreme difficulty. 116 Whether or not deliveries of women who were fat were only occasionally encountered (Giffard, who practised in Brentwood, Essex and Westminster, London recorded only two cases out of 225), it was nevertheless a potential problem for which medical and midwifery books prepared the practitioner. Queen Anne's several stillbirths – of seventeen pregnancies only three children survived birth, though all died later in childhood – may have been perceived, like miscarriage, as not unusual in a woman who was increasingly fat. 117

Labour itself was thought to be harder for a woman who was fat, as it was for those who were contrariwise too thin, or who were experiencing birth for the first time, who were over‐fearful of the birth, and who would not do as they were advised by the midwife during labour. Röesslin told his readers that ‘if the partie be … to[o] grosse and fatte … all such thynges causeth the labour to be much more paynfull cruell and dolorous then it wolde otherwyse be’. 118 The first cause of a difficult and hard labour was also listed by Paré as a fat mother in a chapter on ‘*What the causes of difficult and painful travail in child‐birth are*’. If the ‘fault’ is from the mother it is ‘if she be more fat, if she be given to gormandize or great eating’. 119 Paré here attributes fat simply to over‐eating, a ‘fault’ of the mother's indulgence rather than her constitution, and one that was not a choice for poor women without the means to feed a large appetite. This ‘fact’ was repeated in more or less the same words in later books of midwifery throughout the seventeenth century and into the eighteenth. 120 Such statements of cause suggest that these authors were judging women for their lack of control over their appetites – here literally their appetite for food – in the same way as others generally commented on women's lack of control, over their sexual appetites, their loose tongues and their leaky bodies. 121 To be ‘good’ wives and mothers, fulfilling their reproductive destiny, women should also practise moderation and self‐discipline in diet for their good reproductive health; fat women embodied non‐conformity to gender norms.

The cause of such difficult and painful labours was, as for miscarriage, the pressing down on the internal parts by the fat of the body so that the passage of the child out of the mother's body was obstructed and made too narrow. Guillemeau in a chapter entitled ‘*Of a painfull and difficult Delivery, with the causes thereof*’ remarked upon the obstruction of the passage by large quantities of fat as the cause of a difficult labour: ‘Her person or body may be the cause therof: as if shee be too fat and full: for in such women I have seene great store of fat come down into their naturall parts, which stopped the passage’. 122 As the womb opened and dilated to allow the birth of the child, the omentum with its great store of fat acted as an opposing force, pressing down to shut it again, so obstructing the passage of the child out of its mother's body. The anonymous author of *A Rich Closet of Physical Secrets* (1652) told readers that such women ‘without great help, scarce or never they can part with the child’. 123 Although he did not directly write that fat could obstruct the birth, perhaps taking it for granted that his readers would know what he meant, Maubray in the eighteenth century did repeat what previous authors had said about how to help a fat woman in labour. The midwife was:

to anoint the *Passages* with proper *Unguents*, which ought to be done some time *before*, as well as in the *Hour of LABOUR*: When *she* is likewise to employ her *subtile Hand*, in assisting and augmenting the *Dilatation* of the *Orifice*; as is requisite also in *Case* of the WOMAN being too *Fat* or *Gross*. 124

Not only should the midwife assist delivery by manually stretching open the birth passage, but she should use oil or ointment to lubricate it and so enable the infant to slide out more easily, counteracting the impediment to its birth provided by the fat.

Maubray's advice on how to assist the difficult labour experienced by a fat woman, where the excess flesh of her body would obstruct the passage of the child out of the womb, repeated that offered since Röesslin's *Byrth of Mankynde* in the early sixteenth century:

But and yf the woman be any thynge grosse fat or flesshly it shall be best for her to lye grouelyng for by that menes the matrice is thrust and depressed downewarde anoyntynge also the preuy partes with the oyle of whyte lyllies. And yf necessite require it let not the mydwyfe be afrayde ne asshamed to handle the places and to relaxe and lose the straytes for so muche as shall lye in her for that shall helpe wel to the more expedite and quycke labor. 125

Philip Barrough wrote essentially the same, but put it in slightly different words, 126 while the advice was repeated word for word in *Dr. Chamberlain's Midwifes Practice*. 127 Rueff in *The Expert Midwife* gave further instructions as to how this dilation of the mouth of the womb should be done (‘in bredth only, and not in length at all’), and also that ‘the port‐passage, or outward gate, that is to say, the secrets’ should ‘be more extended, dilated & enlarged’ so that it might ‘better endure all the difficulties of the birth’ to allow the child to come out. 128 The ‘grouelyng’ position to be adopted by the labouring woman stopped the fat of the belly from pressing down upon the womb and birth passage and hindering the birth. Guillemeau, however, also advised that the flesh that was pressing down and obstructing the birth passage should be pushed away and held out of the way by the birth attendant, whom he names as a surgeon rather than a midwife:

If it be, because the Mother is too grosse or fat, and chiefly in her naturall parts, as also if there be any store of fat offer it selfe; (as I have seene it oftentimes happen in great striving and throwes,) yea, and that in such sort, that it did even stop the passage of the childe: Then the Chirurgion (as gently as he can possibly) must thrust backe, and put aside with one hand the said fat, not tearing or hurting it, lest it bee spoiled and corrupted afterwards; holding it still downe on the one side, till the childe be come forth of the wombe, keeping it alwayes from falling downe into the passage, and among the bones, when the childe is ready to come forth. 129

Here Guillemeau was clearly advertising the superior skill of the male surgeon and *accoucheur* in dealing with a difficult birth, and his greater knowledge about the potential dangers of such assistance (the corruption of the handled flesh), as well as his great concern for the comfort and well‐being of the labouring mother, to which he draws attention in emphasizing that he should be gentle in his handling of her body. Lianne McTavish has analysed the stratagems deployed in the writings of French male surgeons as they manoeuvred to gain entry to the previously female‐dominated world of midwifery, presenting themselves as especially sensitive to women's care as well as better able to deal with problematic deliveries. 130 In this discussion of how to deal with the difficult births of fat women, Guillemeau clearly took the opportunity it presented to make the point that surgeons were best placed – most skilled, most knowledgeable, most caring – to assist, overcoming the probable reluctance of women to have their private parts touched – or seen – by a male practitioner. 131 William Sermon in 1671 borrowed this passage from Guillemeau but replaced surgeon with ‘Midwife’, perhaps suggesting it was more appropriate to English circumstances at this time, as midwives still dominated the practice of midwifery in England. 132

However, by the later eighteenth century such repetitions of difficulties in childbirth stemming from the mother's body, and how to assist in these complicated labours, became more infrequent and were replaced with discussion of problems arising from the configuration of the pelvis (‘the bason’), or of the uterus and vagina (such as a weak ‘passive’ uterus or an imperforate or especially narrow vagina). 133 Brudenell Exton's chapter seven, ‘Of *Difficult* BIRTHS’, in his *A New and General System of Midwifery* (1751) opens with the usual statement that difficult births may be caused by mother or infant, but while causes from the infant are the same (size and poor positioning), causes from the mother have changed. Now these include the formation of the mother's pelvis and the situation of her womb: ‘A *Difficult* BIRTH is occasioned either from the oblique Situation, or Largeness of the Child; from the bad Formation of the *Pelvis*, or from the Obliquity of the Womb’. 134 These revised understandings are evident in other works published around mid century, such as in John Burton's *A New System of Midwifry* (1751) where, again, problems from the mother were stated as to do with the pelvis and the situation of the uterus, indicating in this case a shift in obstetrical knowledge and advice from around the mid eighteenth century. 135

Although almost all books of midwifery included some mention of the difficulties caused in pregnancy and childbirth by a woman being fat, those authors who published casebooks listing problematic deliveries did not tend to mention the labouring woman's body in any detail, and when it was referred to, its size was rarely something that was remarked upon. Invariably the expertise of the author in handling the difficult birth was foregrounded and contrasted with those midwives who had mismanaged the birth before his or her arrival. These narratives were both part of a particular practitioner's professional promotion, extolling his or her experience and skill over rivals, but also for surgeons and man‐midwives a means of emphasizing male superiority in the birthing room to justify their incursion into a female‐dominated area of expertise. The labouring woman herself features in these narratives usually only in so far as she is co‐operating with or hindering the birth, to note her pains or lack of them and the breaking of her waters, and any flooding during or after the delivery. This lack of comment may have been because recorded cases were crafted to show off the practitioner's actions, skills and achievements in the birthing room, as historians have previously remarked, but it might also indicate that these practitioners did not often come across women whose bodies were sufficiently different – fatter than usual – to give cause for comment; but when they did, they remarked upon it. 136 Sarah Stone was a midwife who practised in Bridgewater, Taunton, Bristol and probably briefly in London towards the end of her life. 137 She published a selection of her more difficult cases in *A Complete Practice of Midwifery* in 1737, mostly from her Taunton practice, but did not record any difficult births that involved women who were fat. Neither did Edmund Chapman, a man‐midwife in South Halstead in Essex who published fifty case histories in his *Essay on the Improvement of Midwifery* in 1733, and added another seven in a second edition of 1735. 138 However, William Giffard (d. 1731) recorded in his *Cases in Miwifry*, published posthumously in 1734, two cases that commented upon the labouring mother's body as fat and therefore having an impact on the birth. 139 The first of these referred to the fat of the woman's body obstructing and making the birth passage narrower, as midwifery texts had noted would happen.

The majority of the women whose births Giffard recorded were the wives of tradesmen, craftsmen or labouring men who were unlikely to have been able to afford a rich diet and who would have had busy working lives so that they were also unlikely to have been able to over‐eat or to be inactive, leading to excessive weight gain. Authors of midwifery texts noted that working women were less likely to suffer from the deleterious effects of a ‘luxurious’ life, and were therefore better equipped for childbearing. Jane Sharp, for example, advised:

The good order of the body consists in seasonable moderate eating and drinking of wholesome meats and drinks, moderate exercise, for idleness is a great enemy to conception, and that may be the reason that so many City Dames have so few children & if they have any, they are commonly sickly and short lived, it is not so with Country‐women who are always working, they usually have many children, and they are lusty and strong. 140

Robin Ganev has pointed out how these were widespread stereotypes in the eighteenth century in a range of writings from medical to political treatises and popular ballads. 141 There is clearly a social and class dimension to this aspect of early modern perceptions of and attitudes towards fat bodies that cannot simply be explained away by reference to stereotypes about court, city and country. Bodies that were fatter signalled belonging to a higher class where food was more abundant and bodies were not shaped by physical labour. 142 Anxieties about generative difficulties, prompted by concerns about inheritance, may have been more acute in the higher classes, which were also perceived to be more at risk from over‐eating rich foods that were fattening at the same time as they were likely to be less physically active than the labouring population. Fat bodies might feature only rarely in these case histories as they largely documented the labours and births of women from lower social classes: Giffard described these women as poor, or as the wives of barbers, shoemakers, butchers, carpenters, watermen, porters, and so on. 143

In case ninety‐three, ‘*A Delivery in which the Child presented with the Buttocks*’, Giffard recorded that he was called just before midnight on 15 November 1729 by a man who asked him to attend his wife. There Giffard found that the infant was in a poor position for birth and that ‘the Woman's Throws were short, and she could not assist as she ought, because she labour'd under an Asthma and shortness of breath; nor could she bear down so long as she ought to have done’. 144 Giffard turned the child and brought down the legs to deliver it feet first, but the child's shoulders were large and became stuck so that it was with great difficulty that he managed to slowly move them forward and out. Although the child was a live birth, it died soon after. Giffard concluded: ‘its Death, as I judged, proceeded partly from the streightness of the Passage (this Woman was very fat, by which the Vagina was very much stopped up) and partly from the largeness of the Shoulders and Head of the Child, so that it could not be brought out without difficulty’. 145 Here the difficult labour was attributed to both the mother's body and the infant's, with the fat of the mother's body further preventing delivery. Although Giffard did not directly attribute the mother's difficulty in assisting birth, due to her breathing problems and lack of stamina, to the fatness of her body, the reader might have made the connection if familiar with the assertion in other texts that fat people were likely to suffer breathing difficulties.

The high risk of an early death that was associated with fat bodies in both popular and medical writings was explained by their inability to breathe properly: ‘Such haue their vaines very small and close, by reason of their fatnes, that the ayre and breath can hardly haue free course in them, and thereupon the natural heat wanting some refreshing of the aire, is put out, and as it were quenched’. 146 This understanding about fat bodies and the breath continued into the eighteenth century, though now explained in more precise anatomical and medical terms:

But the abundance of it [fat] seldom fails of being attended with *Inactivity* and *Somnolency*, not only from the unwieldiness of an over‐grown Body, nor from the stuffing of the Cavities of the *Thorax* and *Abdomen* alone, which is sometimes so great as to obstruct in some measure the motion and expansion of the *Diaphragm* and *Lungs*, and so to produce a *Dyspnoea* or difficulty of Breathing, and sometimes an *Orthopnoea*, which arises from the pressure of the over‐charg'd *Viscera* of the *Abdomen* upon the *Diaphragm*: But it is probable likewise that the quantity of *Fat* or *Oily* Particles being return'd into the Blood, and engaging and implicating the more subtle active Parts, hinder those Secretions from being made in the *Brain* in that quantity, which is necessary to a vigorous Animal Action. 147

Here the extra flesh of the fat body not only acts again as an obstruction – to the lungs and breathing passages – but also the nature of the fat in the body as an oily substance clogs the blood and interrupts the proper functioning of the body. Such shortness of breath was also particularly associated with pregnant women and the suppression of menstruation, as the blood that was no longer regularly evacuated from the body filled other parts, as Burton explained in the mid eighteenth century:

even in the Beginning of Pregnancy, especially if their Lungs are not good, and if they are subject to Asthmatic Complaints; because, as the *Menses* are suppressed, and no other Evacuation is made, nor the Blood any other way lessened, the Vessels must be filled in every Place: Hence the *Vesiculae Aereae* must be compressed, and cannot easily expand; and at the same Time, the Vessels serving the Intercostals and other Parts of Respiration are oppressed. 148

Furthermore, the ‘*Dyspnoea* or difficulty of Breathing’ that James Drake had identified as a particular issue for those who were fat was also a problem suffered by pregnant women as it would be ‘occasioned by the Extension of the *Uterus*’ pressing upwards to compress the lungs. 149 The French surgeon and *accoucheur* Guillaume Mauquest de la Motte (1655–1737) described such a case in his midwifery treatise published in English translation in 1746 from the original French published in Paris around 1721. La Motte related that he had delivered a woman of four children, ‘who was so short and fat, that the aliments could hardly find room, her stomach being compressed between the *Uterus* and diaphragm’. As a result she was sick a lot if she ate too much ‘and laboured under a very great difficulty of breathing’. 150 In the second case of his delivery of a woman who was fat, Giffard described the labouring woman as ‘very fat and short breathed’. 151 This shortness of breath, in addition to a ‘Navel Rupture’, meant that she was unable to co‐operate in her delivery ‘because she could not retain her Breath, or bear down and assist herself as she ought, whenever her Throws came on’. 152 In this case Giffard directly attributed the shortness of breath to her fatness of body. Like Giffard, La Motte also connected ‘rupture’ or hernia with fat women, reflecting on two cases discussed in chapter six, ‘*Of* delivering *women with* hernia's [*sic*]’, that ‘Both these ladies were very fat, as is usually the case with those that are troubled with this disorder, the *peritoneum* in them being of a more lax contexture and consequently more liable to a dilattion’. 153 Fat women thus laboured with more difficulty, suffering from fat as an impediment to the infant's passage from the womb into the world and from shortness of breath that hindered active participation in pushing the child out.

Connections made between fat bodies and reproductive difficulties, and the concerns expressed about them, are clearly not entirely modern ones. Although early modern writers neither systematically categorized degrees of fatness nor registered differences in fertility and gestational outcomes between women of varying body size (or weight), they detailed how too much fat had a deleterious effect on all aspects of reproduction from sex and conception to childbirth. The sources to which the author has made reference in this discussion represent only some of those that made the same comments and offered very similar advice throughout these two centuries, creating a body of understandings about fat bodies and fertility that was generally consistent and which circulated in both specialist books of anatomy, midwifery, surgery and physick and other popular texts. They perpetuated ideas about fat and fertility stemming from classical medicine and learning that categorized fat bodies as inherently, constitutionally, sub‐fertile, lacking in sexual appetite, and physically unfit for reproduction, including birth. Although identified as by nature, or constitution, problematic in this respect, such bodies could also be brought to a better ‘habit of body’ and would regain their fertility if they could be slimmed down sufficiently (though not too much – too lean bodies were also infertile) through diet and purging, although some practitioners, such as Henry Bracken in the eighteenth century, were not optimistic that this could easily be achieved.

Early modern thinking about fat bodies was thus ambivalent: on the one hand, ‘naturally’ or constitutionally suffering generative difficulty, on the other, liable to alteration through personal remedy because fat was also caused by too much feeding and ill digestion of rich foods from a luxurious appetite. Through examining early modern knowledge and understanding of fat bodies and their generative potential we can gain further insight not only into changes over time in medical and scientific understanding of reproduction and the management of birth (such as from fat as an obstruction to birth to recognition of the importance of the size and shape of the pelvis), but also into continuities in negative representations of fat men and women and how these were perpetuated. The representation of fat bodies as infertile and unfit for sex and motherhood contributed to negative perceptions of them, particularly at a time when fertility and maternity were highly valued. But such representation was also problematic in the questions it raised about class and its relationships with ideals of health and desirability. Lower‐class, labouring bodies were perceived as less likely to be fat and therefore to be more reproductively healthy, more fertile and fit for childbearing, while fatter, potentially less fertile, bodies were associated with higher social status and wealth. Yet in precisely those social classes where production of offspring to continue family lines was regarded as hugely important, that aim could potentially be compromised by the rich living brought by wealth and the display of social status. How thin was the line between pleasingly plump, sexually desirable and fertile and undesirably fat and infertile?

Contemporary concerns about over‐indulgence and luxurious living perhaps owed as much to anxieties about the fertility of the upper classes, and hence about social stability, as they did to moral judgements about gluttony and sloth. These concerns also fed into larger anxieties about women and their lack of control over their bodies and appetites which made them potentially socially disruptive. Women's lack of control over their sexual appetites might give rise to adultery, fornication, prostitution and the bearing of illegitimate offspring, all of which undermined social stability and patriarchal control. Women's lack of control over their appetite more literally might also be socially destabilizing as it could bring generative dysfunction, undermining the primary purpose both of marriage and of women themselves. Men, too, might contribute to their own generative disorders if they did not control their appetite, though to a lesser extent than women who nurtured the growing foetus during pregnancy and brought the infant into the world in childbirth. In doing so they also disrupted contemporary ideals of manhood which encompassed self‐mastery, virility and sobriety. These medical narratives that set out connections between fat bodies, infertility, miscarriage, difficult labours and stillbirths thus also had a regulatory and disciplinary function to optimize early modern bodies for generative success, to secure economic, political and social stability.

It is also clear that modern comprehension of reproductive problems associated with obesity confirms much of what was understood in the early modern world, albeit determined by an entirely different understanding of how bodies worked and how they might become sexually and reproductively dysfunctional. In a society in which fertility was prized, the fact that infertility from fat could be remedied, albeit perhaps with difficulty, meant that the first advice to those fat men and women seeking help for their childlessness was to change their diet and lose fat – advice that would not be at all unfamiliar to a modern practitioner.
